# Matrix metalloproteinase-9 overexpression is closely related to poor prognosis in patients with colon cancer

**DOI:** 10.1186/1477-7819-12-24

**Published:** 2014-01-29

**Authors:** Bo Yang, Fuqiu Tang, Bicheng Zhang, Yong Zhao, Junming Feng, Zhiguo Rao

**Affiliations:** 1 Department of Oncology, Wuhan General Hospital of Guangzhou Command PLA, 627 Wuluo Road, Wuchang District, Wuhan 430070, P.R. China; 2 Second Department of Cadres, Wuhan General Hospital of Guangzhou Command PLA, 627 Wuluo Road, Wuchang District, Wuhan 430070, P.R. China; 3 Department of Pathology, Wuhan General Hospital of Guangzhou Command PLA, 627 Wuluo Road, Wuchang District, Wuhan 430070, China

**Keywords:** Matrix metalloproteinase-9, Colon cancer, Immunohistochemistry

## Abstract

**Background:**

Matrix metalloproteinase-9 (MMP-9) is an important member of the matrix metalloproteinase family and is considered to be involved in the invasion and metastasis of cancer cells. This study analyzed the expression of MMP-9 in colon cancer patients and the relationship between this expression and clinicopathological features and survival.

**Methods:**

We immunohistochemically investigated 68 specimens of colon cancer tissues and corresponding distal normal mucosa tissues using MMP-9 antibody. Then, the correlation between MMP-9 expression and clinicopathological features and its prognostic relevance were determined.

**Results:**

The expression rate of MMP-9 in colon cancer tissues was significantly higher than that in distal normal mucosa (69.1% versus 2.9%, *P* < 0.001). Significant correlations were only found between high levels of MMP-9 expression and metastasis of lymph nodes and Dukes’ stage. Overexpression of MMP-9 was associated with shorter survival times in univariate analysis. Multivariate analysis confirmed that MMP-9 expression was an independent prognostic factor.

**Conclusions:**

MMP-9 is correlated with the metastasis of lymph nodes, and its elevated expression may be an adverse prognostic indicator for the patients of colon cancer.

## Background

Colon cancer is the most frequent digestive system cancer in the world. It is also the third most common malignant tumor in the United States. In 2012, the estimated new cases of colon cancer were 103,170 throughout the country [1]. With the development of economy and the changes in dietary patterns, the incidence of colon cancer is also increasing rapidly in China. Now, colon cancer is the fourth most common malignant tumor and the third leading cause of cancer death in Chinese people [2].

The invasion and metastasis of cancer cells always result in treatment failure. Extracellular matrix (ECM) degradation is an important stage of tumor metastasis, which is regulated with matrix metalloproteinases (MMPs) [3,4]. The MMPs carry out the selective proteolytic degradation of ECM, which is an imperative step for the migration and invasion of tumor cells. MMPs are divided into six categories according to the specificity of their substrates as follow: interstitial collagenases, gelatinases, stromelysins, matrilysins, membrane-type MMPs and others [5]. MMP-9 is an important member of the gelatinases. It is also called gelatinase B or 92 kDa type IV collagenase [6]. The gene of MMP-9 is located in 20q11.2-q13.1. MMP-9 can be involved in the development of several human malignancies, as degradation of collagen IV in the basement membrane and the extracellular matrix facilitates tumor progression, including invasion, metastasis, growth and angiogenesis [7].

However, the correlation between MMP-9 expression and survival or prognosis in colon cancer is still inconclusive. Here, we immunohistochemically investigated 68 specimens of colon cancer tissues and corresponding distal normal mucosa tissues. Then, the association of MMP-9 expression with clinicopathologic features and prognosis was analyzed by univariate and multivariate analysis. The results of this study could provide new evidence for the research of MMP-9 in colon cancer.

## Methods

### Ethics statement

The study was approved by the Wuhan General Hospital Ethical Committee. Written informed consent was obtained from all participants.

### Patients and tissue samples

Colon cancer tissues and corresponding distal normal mucosa tissues of 68 patients who were treated at Wuhan General Hospital of Guangzhou Command PLA between January 2005 and January 2007 served as the study material. Diagnosis and staging were performed according to the modified Dukes’ classification [8]. Forty-four patients were men and 24 patients were women. The mean age was 58 years with a range of 47 to 70 years. The depth of invasion was classified as mucosal and submucosal membrane layer, muscular layer and serosa layer. Tumors were classified as well, moderately and poorly differentiated adenocarcinomas. Lymph node metastasis happened in 27 patients, and the other 41 patients had no metastatic lymph nodes. All patients were followed up for survival. None of the patient underwent radiotherapy or chemotherapy before surgery. Formalin-fixed and paraffin-embedded surgical tissue samples were collected from the archives of the Department of Pathology, Wuhan General Hospital of Guangzhou Command.

### Immunohistochemistry

The paraffin-embedded colon cancer tissues and corresponding distal normal mucosal tissues were cut at 4 μm and mounted on glass slides. Then, the slides were dewaxed in xylene and rehydrated in ethanol, and treated with a solution of peroxidase-blocking reagent (Dako, Glostrup, Denmark) to exhaust endogenous peroxidase activity. They were put in 0.01 mol/L citrate buffer at pH 6.0 for 15 minutes in an 800 W microwave oven and then left at room temperature for 20 minutes to expose antigen hidden inside the tissue due to formalin fixation. To inhibit nonspecific antigen-antibody reactions possible in immunohistochemical staining, protein blocker (Research Genetics, Huntsville, AL, USA) was used for 5 minutes, and the slides were washed thoroughly with PBS buffer. Then the slides were incubated overnight with the primary antibodies against MMP-9 (1:500; mouse monoclonal antibody, sc-21733, Santa Cruz Biotechnology, Inc., Santa Cruz, USA) at 4° centigrade. Biotinylated goat anti-mouse secondary antibody (1:200; BA1001, Boster Bio-engineering Limited Company, Wuhan, China) was applied for 20 minutes at room temperature, followed by further washing with buffer to remove unbound antibody. A complex of avidin with horseradish peroxidase was then applied for 20 minutes at room temperature. For color development, the slides were stained with 3,3′-diaminobenzidine tetrahydrochloride (DAB, Sigma-Aldrich, St Louis, MO, USA), then counterstained with hematoxylin. A reddish brown precipitate in the cytoplasm of cells indicated a positive reaction. In each immunohistochemistry run, the positive section provided by the reagent company served as the positive control, and omission of the primary antibody served as negative control. Immunohistochemistry stained slides were reviewed by two investigators independently blinded to all clinical data. A scoring system was used to describe both intensity of staining (0, negative; 1, weak; 2, moderate; 3, strong) and the percentage of cells stained (0, 0%; 1, 1% to 5%; 2, 6% to 75%; 3, 76% to 100%). The final score was determined by the combined staining score (extent + intensity) [9]. Score ≥3 was defined as positive expression.

### Statistical analysis

MMP-9 expressions in colon carcinoma and normal mucosa were compared by χ^2^ test. The χ^2^ test was also used to examine MMP-9 expressions in various clinicopathological characteristics. The univariate survival analysis and cumulative survival curve were executed by Kaplan-Meier method. The difference between the curves was analyzed by Log-rank test. The multivariate survival analysis was executed by Cox proportional hazard regression model. A *P* value < 0.05 was considered statistically significant. All statistical analyses were performed with *SPSS* 13.0 (SPSS Inc., Chicago, IL, USA).

## Results

### Matrix metalloproteinase-9 expression in colon carcinoma and distal normal mucosa

**Figure 1:**
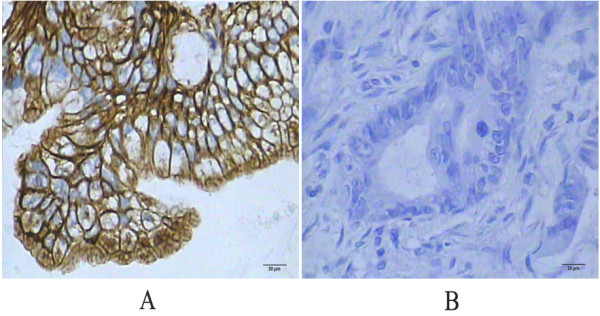
**Matrix metalloproteinase-9 (MMP-9) expression in colon cancer tissues. A**: Positive MMP-9 expression in colon cancer tissue. The expression of MMP-9 was detected in the cytoplasm by immunohistochemistry staining (×400, SP method). **B**: Negative MMP-9 expression in colon cancer tissue (×400, SP method).

Positive MMP-9 expression was observed in 69.1% (47/68) of the colon cancer tissues, and in 4.4% (3/68) of the distal normal mucosal tissues (Figure 1). The expression of MMP-9 was detected in the cytoplasm. The difference of MMP-9 expression between colon cancer and distal normal mucosa was statistically significant (χ2 =64.602, *P* < 0.001).

### Correlation of matrix metalloproteinase-9 expression and clinicopathological features in colon cancer

When comparing the MMP-9 status with clinicopathological variables, we found significant positive correlations between MMP-9 expression and lymph node metastasis (*P* = 0.020), and Dukes’ stage (*P* = 0.029) (Table 1). Kaplan-Meier analysis showed that the differences of survival in the MMP-9 expression group and the Dukes’ stage group were highly statistically significant (Log-rank test, 11.010, *P* = 0.001; Log-rank test, 11.831, *P* = 0.001). Meanwhile, the differences of survival in metastasis of lymph node groups and infiltrative depth groups were also statistically significant (Table 2). Importantly, there were no significant differences between the three groups in terms of patient age, gender and tumor differentiation. The MMP-9 expression appeared as a significantly independent prognostic factor (*P* = 0.008) with a relative risk of 1.857 (95% confidence interval, 1.117 to 3.088) in Cox multivariate analysis, which was done with the following variables for each case: MMP-9 expression, metastasis of lymph node, infiltrative depth, and Dukes’ stage (Table 3).

**Table 1:** The correlation of matrix metalloproteinase-9 (MMP-9) expression and clinicopathological features in colon cancer patients

**Items**	**Cases**	**MMP-9**		
		**Positive cases**	**χ2**	***P***
Gender				
Male	44	32	0.761	0.383
Female	24	15		
Age				
<58 years	32	21	0.345	0.557
≥58 years	36	26		
Tumor size				
<5 cm	41	27	0.515	0.473
≥5 cm	27	20		
Differentiated degree				
Well differentiated	32	23	1.144	0.565
Moderately differentiated	19	14		
Poorly and undifferentiated	17	10		
Infiltrative depth				
Mucosa and submucosal layer	11	5	5.872	0.053
Muscular layer	24	15		
Serosa layer	33	27		
Metastasis of lymph node				
Negative	41	24	5.416	0.020*
Positive	27	23		
Dukes’ stage				
A	11	4	9.051	0.029*
B	30	20		
C	17	14		
D	10	9		

*Statistically significant.

**Table 2:** Results of survival analysis for individual factors

**Factor**	**Log rank**	***P***
Matrix metalloproteinase-9 (MMP-9) expression	11.010	0.001*
Gender	0.520	0.471
Age	0.041	0.839
Tumor differentiation	0.055	0.814
Metastasis of lymph node	8.687	0.003*
Infiltrative depth	6.141	0.013*
Dukes’ stage	11.831	0.001*

*Statistically significant.

**Table 3:** Results of Cox multivariate regression factors

**Factor**	**Wald value**	***P *** **value**
Matrix metalloproteinase-9 (MMP-9) expression	5.698	0.017*
Metastasis of lymph node	0.378	0.539
Infiltrative depth	0.159	0.690
Dukes’ stage	6.597	0.010*

*Statistically significant.

### Relationship between matrix metalloproteinase-9 expression and survival rate of colon cancer patients

**Figure 2:**
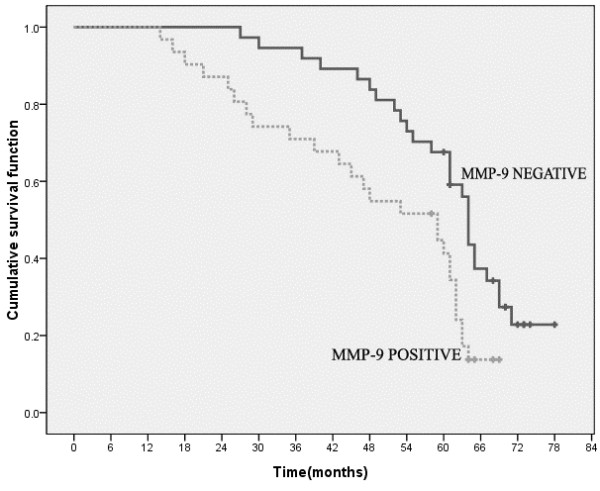
**Kaplan-Meier survival plots of matrix metalloproteinase-9 (MMP-9) positive group and negative group.** There is highly significant difference in survival rate between the MMP-9 negative group and the MMP-9 positive group (Log-rank test = 11.010, *P* = 0.001).

The 5-year survival rate for 68 colon cancer patients was 57.4%. The 5-year survival rates for MMP-9 negative and MMP-9 positive cases were 67.6% and 41.9%, respectively. The difference of survival rate between the MMP-9 negative group and the MMP-9 positive group was statistically significant (Log-rank test = 11.010, *P* = 0.001) (Figure 2).

## Discussion

Colon cancer is one of the most familiar malignant neoplasmas. The pathogenesis of tumor is a process of multiple factors, multiple steps and many stages, which are concerned with the abnormalities of many oncogenes, tumor suppressor genes, mismatch repair genes and cellular adhesive factors [10]. But the invasion and metastasis of tumor cells were main causes for cancer treatment failure among these factors. MMP-9 is the most complex member of the MMPs family in terms of domain structure. It is capable of degrading decorin, elastin, fibrillin, laminin, gelatin, and types IV, V, XI and XVI collagen [11,12]. The expression of MMP-9 was regulated by many upstream factors. Levels of phosphorylated signal transducer and activator of transcription 3(STAT3) regulated the MMP-9 gene in pediatric patients with ulcerative colitis [13]. Ursolic acid (UA), a natural pentacyclic triterpenoid carboxylic acid distributed in medical herbs, also suppressed colon cancer cell migration by inhibiting MMP-9 expression [14]. Increased CO_2_ concentration also elevated the mRNA expression of MMP-9 and invasive capability in colon cancer cell lines and human samples derived from a peritoneal metastasis [15]. Knockdown of metastasis-associated in colon cancer 1(MACC1) expression using shRNA reduced hepatocellular carcinoma Huh7 cell migration and invasion abilities, which were associated with the downregulation of MMP-9 protein [16]. Detected by using a luciferase reporter construct and western blots, piwi-like protein 2 (Piwil2) may regulate a 2-kb MMP-9 promoter fragment and apoptotic pathways in colon cancer [17].

Overexpression of MMP-9 has been found to associate with the invasion and metastasis of cancer [18]. The level of MMP-9 expression showed a statistically significant correlation (*P* < 0.001) with the disease histopathologic grade, stage, metastatic potential, recurrence potential, and survival in patients with squamous cell carcinoma of the larynx. The Kaplan-Meier curve linearly showed the MMP-9 expression as a predictor of survival to be significantly (*P* < 0.001) associated with survival [19].

The increased MMP-9 expression makes the main contribution to the invasive potential of squamous cell cervical carcinomas [20]. Elevated serum MMP-9 correlated with distant metastasis and poor survival in patients with squamous cell carcinoma (SCC) over either the head and neck or the esophagus [21]. Elevated serum MMP-9 level was also associated with reduced disease-free survival (DFS) of breast cancer [22].

In this study, we found that MMP-9 expression in colon cancer tissues (47/68, 69.1%) was significantly higher than that in corresponding distal normal mucosa tissues (3/68, 4.4%), and there was a statistically significant difference (χ2 =64.602, *P* <0.001) between them. Furthermore, high levels of MMP-9 expression in colon cancer cells correlated with lymph node metastasis and with Dukes’ stage. Therefore, these findings suggested that MMP-9 was likely to play a role in promoting tumor invasion and metastasis. Meanwhile, Kaplan-Meier analysis showed that the differences of survival in metastasis of lymph node groups, infiltrative depth groups, MMP-9 expression group and Dukes’ stage group were highly statistically significant. Cox multivariate analysis suggested that MMP-9 might serve as an independent marker for poor prognosis. Unsal D *et al*. reported that MMP-9 expression was characterized by poor overall survival and DFS in patients with Stage II/III rectal carcinoma [23]. Here, our results showed that MMP-9 might be correlated with the metastasis of lymph node, and its elevated expression might be an adverse prognostic indicator for the patients of colon cancer. Although the detailed molecular mechanism involved in this process is less well defined, this study still has potential clinical benefits. The MMP-9 expression that could be detected by immunohistochemistry may be a useful molecular marker to predict the prognosis in colon cancer patients.

## Conclusions

In conclusion, our study suggests that MMP-9 plays an important role in invasion and metastasis of colon cancer, and thus becomes a useful indicator for clinical assessment of tumor biological behavior and prognosis in colon cancer patients.

## Abbreviations

DAB: 3,3′-diaminobenzidine tetrahydrochloride; DFS: disease-free survival; ECM: extracellular matrix; MACC1: metastasis-associated in colon cancer 1; MMP-9: matrix metalloproteinase-9; MMPs: matrix metalloproteinases; Piwil2: piwi-like protein 2; SCC: squamous cell carcinoma; STAT3: signal transducer and activator of transcription 3; UA: ursolic acid.

## Competing interests

The authors declare that they have no competing interests.

## Authors’ contributions

BY drafted the manuscript. JF and ZR designed the study and helped in drafting the manuscript. FT, BZ, and YZ collected the data and performed the statistical analysis. All authors have read and approved the final manuscript.
